# Foreign Summary

**Published:** 1839-04-13

**Authors:** 


### FOREIGN SUMMARY.

*Professor Grant on the Infusoria.*—Dr. Grant entertained the visitors this evening at the Royal institution, with a short account of the recent researches into the nature of the *infusoria.* He explained the import of the same by stating that the attention of philosophers was first directed to them as a product of putrescent infusions. The microscopic investigations of Liewenhoeck had thrown great light on the anatomical structure of these animalcules; and successive entomologists, but pre-eminently Ehrenberg, had made us acquainted with the almost universal presence of these creatures in nature. They were shown to be necessary inmates of all fluids; rivers and the profound ocean derived colour from them. The persons of men and the bodies of all animals were menageries for their entertainment. They had been found in the deepest mines yet penetrated, though totally removed from the influence of the sun's rays. They had been found (said the Doctor) in dried mud, in a state of suspended animation, and constituted a large portion of the clouds of dust which are transported by the winds over the earth's surface. Thus dry and torpid, and strown over the ground, by returning moisture they are restored to a state of active function. They are to be met with in all parts of the world, and their locality is not regulated by any distributory laws. They subsist in a state of hybernation within the arctic circle, and there are five or six hundred species of them existing in the sands of the African desert. The silicated kinds remain unscathed, even in sulphuric and muriatic acids. The imperfection of our present knowledge regarding them, prevents our forming an accurate classification of them; but Dr. Grant considers the lines of their organization, as drawn from East to West, more homogeneous than any that can be drawn from North to South. The *débris* of these animalcules constitutes an immense portion of the stratified rocks. The masses of flint found in the beds of the earth, are but agglomerations of defunct silicious infusoria, and the immense hills of chalk are supposed to be merely the exuviæ of similar animalcules. There can be no doubt, according to the lecturer, that these minute monads have been the architects employed in modifying the surface of the globe, and preparing it as a residence for man.

Dr. Grant divides these animalcules into two large classes—the wheel animalcules, or *rotiferæ*, and the more simple polygastric monads. The wheel infusoria are of complicate structure. They are supplied with œsophageal ganglia, and with organs of respiration, digestion, &c., rivalling in complexity and completeness a much higher grade of the animal kingdom. On this occasion he confined his attention chiefly to the polygastrica. Lamarque and one or two other naturalists thought that these animals were nourished by endosmosis, and that their movements were automatous, and not spontaneous; but the more accurate observations of Liewenhoeck, Spallanzani, Goets, Walp, Deicken, and Ehrenberg, have shown that they possess not only an alimentary canal varying in direction, but a masticatory apparatus. Ehrenberg's ingenious and elegant devices have constituted the most successful means of developing the structure of the polygastrica. Their immersion in coloured but innocuous infusions has pointed out the existence of a plurality of digestive pouches, whence their designation is derived. Many of these monads are of the most voracious character, and devour whole hecatombs of the inferior species. The Doctor demonstrated the structure of these creatures by a profusion of drawings and diagrams, in which the outward form, internal splanchnology, and mode of propagation, were most satisfactorily illustrated; such as of the *monus crepuscula*, the *valora globator*, &c., of which it would be impossible to give an adequate idea in letterpress. Thousands of species are parasites of the conferbæ, polevaceæ, &c.; whilst others, as has been said, are antovorans. But nothing connected with these animalcules is more astonishing than their propagatory powers. Their mode of generating is of two kinds, oviparous and *fasciparous;* and in many species both modes of propagation are in active operation. In numerous instances these monads spontaneously divide themselves into two, others into four segments, each of which is as perfect a being as the parent. It is known that several of the species propagate by this means at the rate of 124 millions in five hours. But such as propagate in both fashions are calculated to produce, in forty-eight hours, 120 millions of billions; or in other terms, one of these living specks which a strong microscope alone can render visible to the human eye, can, in 48 hours, magnify or multiply itself to a dimension of two cubic feet—a stupendous example of vital energy to which any thing visible in the higher animals is indeed feeble in comparison. These monads agglomerate together into immense communities, which exhibit various forms of contour—globular, oblong, or circular; and the mausolea of many of these defunct nations remain extant at this day in the shape of masses of flint. A new view of the nature of the ponipherous classes of animals had recently been promulgated in a memoir read before the Institute of France, in which it is contended that the surfaces of the sponge are covered with innumerable distinct and independent monads. This theory Dr. Grant thought exceedingly probable.—*Medical Gazette.*

---

*Scirrhus of the Œsophagus, (two cases.) With Remarks.* By Professor GRAVES.—The two following cases were in our wards at the same time, and afforded a good opportunity of comparing together the symptoms observed in each. In one, Benjamin Spear, we for a long thought that the difficulty of swallowing was spasmodic, so completely was the power of deglutition restored (and that, as will be seen from the notes of the of the case, for many days) by passing an œsophagus bougie into the stomach. In the other, Thomas Berry, the patient could at all times swallow liquids with great facility. He was able to drink a tumbler of water with as much apparent ease as any healthy person; but soon after gulped up the fluid by mouthfuls; as the fluid passed readily into the stomach, and was only rejected after arriving there, the diagnosis was rendered very obscure, and I attributed his sufferings to disease of the stomach itself. On this account a trial was not made with the bougie, except once before the man's admission, by Mr. M. W. Murphy, the practising pupil who had the care of this patient while in the Hospital, a gentleman, to whose untiring industry and ability I have been frequently indebted for valuable observations. Mr. Murphy did not succeed in passing the bougie, but as he never before attempted this operation we did not attach a proper degree of credit to this trial.

Altogether I should hope that the account given of the symptoms and post mortem examinations of these two patients, will prove useful in elucidating the diagnosis of stricture of the œsophagus. These cases afford another example of the fluctuating, or even contradictory nature of certain symptoms in different individuals affected with the same disease. It is essentially necessary for the physician to be aware of this circumstance, for it teaches him, that in endeavouring to make out the true nature of any affection, he must refer not to a fixed, but a varying standard of comparison. Whether these variations, in the two following patients, could be accounted for by any differences in the diseased parts observed after death, must be left to the judgment of the reader.

Thomas Berry, æt. 64; admitted September 23d; ill four months. He states that he had always been temperate and healthy, and that about five months ago he was attacked, after exposure to cold, with cough, without expectoration, pain in the side, or any other symptom for about a month, when he experienced a slight pain at the ensiform cartilage, which generally came on after eating; this continued every day, becoming more severe for five weeks, and he then experienced a difficulty of swallowing, which he referred to the seat of pain, where he says his food always stopped for about two seconds, and was then rejected. These two symptoms, viz. pain at the ensiform cartilage, and inability to retain food, have every day become more distressing, and are the only things of which he complains; took no medicine before admission.

*Present State.*—Extreme emaciation, and great debility, having eaten scarcely any thing for the last two months, being quite unable to retain either solids or fluids; the latter pass without much difficulty into the stomach, and remain there for about half a minute, but are then gradually gulped up, apparently without an effort. The cough has been very troublesome for the last few days, accompanied with abundant mucous expectoration. Never vomited any black matter, or any thing except what he swallows.

Bowels have been costive since his illness commenced; frequently for eight days without a motion; appetite good; pulse 54; abdomen fallen; no tumour to be felt; the skin is shrivelled and dry, its elasticity quite impaired; tongue clean and moist; skin cool; sleeps tolerably.

Extr. Conii granum, Syrupi, Mucilaginis, aa q. s. ut ft. bolus. Quarter in die sumendus.

26th. Was able to retain the bolus, and also a small quantity of broth; feels improved; pain and tenderness in epigastrium diminished; urine high coloured.

27th. Cough very troublesome, preventing sleep; abundant sero-mucous expectoration; no change in the other symptoms.

Vesicat. abdom. Pulv. Conii gr. ii. ter die.

29th. The blister did not rise, though left on for twenty-four hours; milk now remains on his stomach, but a solid is immediately rejected. Complains of great pain in the epigastrium, where there is also considerable tenderness. He says he knows by the sensation which the food produces when going down, whether it will be rejected or not, and he so accurately foretells this, that many suspect he has a power of bringing it up when he pleases. When it is to come up it excites a kind of spasm, from which he seems to suffer much; pulse 70; cough very troublesome; expectoration copious, yellow mucus, mixed with a great deal of serum: no rale over any part of the chest.

Sinapismus abdomini.

30th. Sinapism produced no effect; took some tea and whey yesterday, which he immediately rejected, and was shortly after attacked with a severe pain about the false ribs, which he attributes to the straining; this with the cough kept him awake the greater part of the night.

Turpentine stupe, and afterwards a sinapism.

October 1st. He states, that yesterday evening he felt that "*his swallow had returned*," and that his "*stomach was opened*," and immediately ate a large bowl full of stirabout and milk, all of which he was enabled to retain on his stomach. Bowels opened once; all his symptoms are aggravated when the bowels are confined; acetic solution of cantharides to be rubbed to the abdomen.

3d. The last application caused vesication, and he is to-day much improved, and can retain solids as well as fluids.

Repr.

6th. Ate some bread yesterday evening, but was unable to retain it, and has since frequently vomited ; cough troublesome ; complains of pain about the false ribs, also in the epigastrium, which is still tender ; tongue moist.

Acidi Hydrocyanici medicinalis gutts. iii. ter in die.

24th. No material change since last note : one day he could retain his food, and the next would be unable to do so. Last night was attacked with severe pain in the right false ribs, which prevents him from taking a full breath : troublesome cough ; copious expectoration. The whole of the right side is so tender that he cannot bear the slightest pressure : great thirst ; tongue furred and moist ; pulse 56.

26th. The pain last night became so severe in the right side that it caused a kind of convulsion, during which he worked violently for two hours. Tongue furred and moist ; great thirst ; can scarcely speak ; extreme debility ; has not eaten any thing for the last three days.

Died on the 27th.

*Autopsy eighteen Hours after Death.*—The abdomen was considerably distended, though before death it was remarkably collapsed, and tensely concave. On opening the abdomen the stomach and intestines were found distended with air ; and in the latter were hardened fæces. On raising the stomach, the coats were so thin, and so much softened, that the fingers passed through them in every direction ; the mucous membrane was very soft and easily detached. The last two inches of œsophagus were inflamed ; and above this, to the extent of about three inches, was a continuous mass of scirrhous growth, contracting the œsophagus to about the size of a goose-quill ; the mucous membrane above this was thickened and softened, and could be easily separated from the submucous tissue.

Left lung healthy ; the right was connected by strong adhesions to the parietal pleura, in the cavity of which was found nearly a pint of thin fluid, mixed with shreds of lymph, (recent;) the lower portion of the lung was covered with lymph ; spleen enlarged and very soft. Two of the vertebræ opposite the structure, presented knobs on their anterior surface. These knobs projected about three-quarters of an inch beyond the remaining surface of the vertebræ ; they were covered with a thin lamina of bone externally, and they displayed a healthy cancellated structure, continuous with the cancellated tissue of the vertebral bodies. They consisted, therefore, of an exuberant growth of healthy bone, and they each comprised a portion of two contiguous vertebræ. The intra-vertebral substance had undergone a corresponding increase, and was prolonged so as to divide each knob into two portions. It could not be ascertained whether these bony protuberances had any connexion with the production of the stricture. In this case the vomiting or rejection of the food, *after it had passed the stricture*, was a very remarkable circumstance ; it may, perhaps, be explained by supposing that the inflammation of the œsophagus extended to the stomach. The stomach was excessively thin and membranous ; in fact it was, like all the muscles of the body, extremely emaciated. This case was watched and recorded by Mr. James Brady, one of our most distinguished pupils.

The scirrhous mass was in this man rather considerable, and had caused a nearly complete degeneration of all the tissues of the œsophagus. Posteriorly, where it was thickest, it was three-quarters of an inch in depth, and it had evidently arrived at the stage next to that of ulceration : it was not yielding or elastic. These circumstances account not only for the narrowness of the stricture, but for the inflammation of the mucous membrane of the stomach and œsophagus ; on this account too the bougie would not pass.

In all these particulars it forms a strong contrast with the next case, where the morbid tissue was still elastic, and the stricture dilatable and free from inflammation.

Benjaman Spears, æt. 50, admitted into hospital August 29th, 1838. Had been a soldier, and and served many years in the East Indies ; of most intemperate habits. Says he has been always healthy, never having jaundice or ague ; never subject to cough or dyspnœa. Says that about a month since, he noticed a slight soreness on swallowing, referred to epigastric region, which continued for four or five days ; when on attempting to swallow a piece of bread, he found it stop at a part corresponding to about the centre of the ensiform cartilage, and that he immediately rejected it ; that since then he has been unable to retain any thing ; that on its passing down, it is rejected in a few seconds without any effort ; has taken nothing for three weeks. Bowels have been confined ; had one movement each week ; appetite has been bad, and his sleep much disturbed ; has not had cough or pain in the chest.

*Present state*, August 30th.—Great emaciation; countenance sallow, and anxious ; abdomen fallen ; total inability to retain either solids or fluids. Feels, on attempting to swallow, a pain at inferior part of ensiform cartilage, to which he refers the obstruction ; the food is returned without any effort, the diaphragm scarcely appearing to act. On measuring the quantity swallowed, and after its being rejected, it is found increased, appearing to be more than the addition of the saliva would produce. Some tenderness on pressure in epigastrium and right hypochondrium ; has no pain elsewhere ; no tumour ; *no dyspnœa or cough ;* much thirst ; tongue dry and slightly coated. Bowels confined ; extremities cold ; pulse 100, very feeble and small ; respiration 15, natural ; on deep inspiration feels some soreness in right hypochondrium.

℞. Solut. Ichthyocol. ℥iii.
Tincturæ Opii. gutts. v.
Ft. Enema bis in die injiciendum.
Applic. Emp. Lyttæ Epigast.

The œsophagus bougie to be passed.

31st. Œsophagus bougie passed yesterday by Dr. Collis, *who says he met with no obstruction:* immediately after the passing the bougie, felt some water which he took pass beyond the obstruction; has taken some whey since, had slight nausea on swallowing it, but it remained.

31st. To get a pint of isinglass and milk.

September 1st. On attempting to swallow a small piece of meat yesterday, felt considerable pain, and rejected it immediately. Is able to swallow and retain the isinglass and milk; is greatly better.

4th. Has had no vomiting since; has taken the isinglass and milk regularly. Bowels are confined; has had no cough; was seized yesterday with a severe stitch in right side, under mamma, attended with dyspnœa.

7th. Had vomiting yesterday, but was able to retain some of his milk; is very weak; the pain in side better; very little cough. Tongue dry; pulse 76, very feeble.

9th. Total inability to swallow; every thing rejected; refers the obstruction to same place as before; pain in side better. Pulse 80; no cough. Bougie passed without difficulty.

10th. *Retained every thing after passing the bougie;* has much headache; the tenderness of the epigastrium nearly gone; pain in side better.

11th. Was seized with severe pain in right infra-mammary region last night, with much dyspnœa and cough; had no vomiting since.

12th. Pain still very severe; much cough; expectoration scanty; no vomiting.

13th. Pain still very severe; much cough; no vomiting.

14th. Pain still severe, preventing him from sleeping; had no vomiting.

18th. Pain in side still continues; is very weak; cough troublesome; sputa very abundant; *no vomiting.*

25th. In same way; *no vomiting;* expectoration profuse; pain less severe.

30th. In same way.

October 8th. Mentioned that he had a swelling in perinæum, which was opened by Mr. Crampton, and a large quantity of very fœtid, thin matter discharged, from which he found great relief. Cough very severe; expectoration very abundant.

12th. Very weak; continues in same way; cough severe; expectoration profuse, of same character as before.

18th. Expectorated in night a large quantity of puriform matter, very fœtid; is excessively weak; pulse 100, feeble and thready; extreme emaciation. Examined in infra-mammary region of the right side corresponding to seat of pain, a distinct gargouillement, with cavernous respiration, was for first time audible; pectoriloquy partial; extremities cold.

Died at three o'clock, on 19th.

*Autopsy.*—Appearance of body extremely emaciated. On opening the œsophagus, all its upper part was found quite healthy, to within three and a half inches of its termination, where a stricture existed, through which the little finger could not be passed, but which admitted a large metal bougie, one-quarter of an inch in diameter. On slitting open the structured parts, the mucous membrane appeared quite healthy, without any appearance of ulceration; and on dissecting the mucous coat off, the stricture was found to arise from a deposite of a cartilaginous structure in the circular fibres of the muscular coats, which, as well as the longitudinal ones, were exceedingly thin and scarcely to be distinguished; the deposit was irregular, being thicker in one part than another. The stricture was an inch and a half in length; the mucous glands above the stricture were somewhat enlarged; the stomach healthy, but contracted: and the intestines presented no morbid appearance. Strong adhesions attached the right lung to parietes, which on being torn through the fingers passed into a large superficial cavity of irregular depth, corresponding to the infra-mammary region, where the acute pain was complained of. Several crude tubercular deposits existed in different parts of the lung, but none of them in a state of softness; several small calcareous bodies were found in the apex of same lung: left lung was quite healthy.

In this case the stricture was easily dilated; and the operation of passing the bougie produced such remarkable and so long continued relief, that I was led to consider the obstruction as merely spasmodic, induced by a passing or temporary œsophagitis.—*Dublin Journal.*

---

*Remarks on Burns.* By Samuel Cooper.—That many persons who meet with burns die comatose, or else with great difficulty of respiration—asthmatic symptoms, as they were called—were facts well known to surgeons many years ago. The cause of coma was not, however, attempted to be explained, as it might correctly have been, by reference to the congestion of the vessels of the brain, and the effusion upon or within that organ, as subsequently demonstrated in post-mortem examinations; while the old practitioners, instead of looking at the congested and even inflamed lungs, by which they would have been able to account rightly for the oppression of the breathing, ascribed the latter frequent consequence of a burn to sympathy between the lungs and the injured skin. This was the doctrine which I used to hear inculcated by Abernethy.

The post-mortem examinations made by Dupuytren, of individuals who died of burns, threw quite a new light upon the subject. They proved, that when the sufferer perishes in the flames, or shortly after being removed from them, marks of excessive congestion are usually observable in the intestinal canal, although there has not been sufficient time for inflamation to commence. Not only does the mucous membrane exhibit bright red patches—not only is it gorged with blood, but the bowels contain a quantity of this fluid, which has been extravasated. He describes the brain as being largely injected with blood, and the fluid in the serous cavities of the body as presenting a reddish colour. He represents the mucous secretion of the bronchial tubes as also bloody, and their investing membrane as exhibiting a bright red colour, and streaked with highly injected capillary vessels. It seemed to him as if the blood, suddenly repelled from the skin, made an effort to escape through the pores of every internal surface.

Our second case exemplifies the truth of most of these observations, with the exception that *the mucous membrane of the bowels was pale*, though the lungs and brain were much congested, and a bloody serous fluid was copiously effused within the cranium and the chest.

Dupuytren found that, if the patient died between the third and eighth days after the receipt of the burn, traces of inflammation of the bowels, lungs, and brain, were commonly noticed; but if the patient sank at a later period, or in the suppurative stage, the mucous membrane of the intestines was generally studded with patches of redness and ulceration, and that sometimes the mesenteric glands were enlarged.

As we have not met with such enlargement of the mesenteric glands in our post-mortem examinations of burnt patients, a doubt is left in my mind whether such enlargement, as remarked by Dupuytren, depended upon the burn, or upon the effects of scrofulous disease existing previously to the accident.

The entire perforation of the duodenum by ulceration, exemplified in our first case; the adhesion of the margins of the ulcerated opening to the pancreas; the discharge of great quantities of blood from the rectum before the patient sunk; and the blood found after death within the intestinal canal, and, no doubt, the source of which was the considerable ulcer in the duodenum; appear to me to be circumstances all deserving to be well remembered.

The vomiting, in our second case, first of a brown fluid, and, as early as the sixth day, of blood; the death of the patient at the end of the first week; the presence of several ulcers in the duodenum at this early date; its actual perforation in one place by the ulcerative process; and the presence of blood in the stomach, duodenum, and ileum, after death, are so many facts of great interest in relation to the pathology of burns. Dupuytren's observations would not lead us to expect ulceration of the bowels so early. As for the vomiting of blood, and its discharge *per anum*, I am not aware that he has adverted to these occasional consequences of burns at all.

Our last case, besides exemplifying several effects arising from visceral inflammations, presents us with an instance of an ulcer of the mucous membrane of the stomach nearly cicatrized.

These post-mortem investigations seem to me, gentlemen, not only to elucidate the causes of various symptoms, observed to follow burns, but to suggest the question, whether, in the stages of burn, attended with congestion, or actual inflammation of important internal organs, the taking away of blood from the patient would not be the most likely means of saving the patient's life. In France, I know that the use of leeches, in certain stages of burns, is advocated by some surgeons, as much as they are by certain practitioners here, in the commencement of an attack of erysipelas. In the period of reaction, between the third and eighth days, when the pulse is strong, and there is evidence of visceral inflammation having come on, what measure is so likely, I ask again, to save the patient? Let the result of a moderate abstraction of blood be first ascertained; and, if it be favorable, let the evacuation be repeated with circumspection.—*London Med. Gaz.*

---

*Extract of Taraxacum.*—Generally this has a sweet taste, and is readily soluble in water; but Mr. Squire, who has paid much attention to this and other extracts, informs me that, when cautiously prepared, and not unnecessarily exposed to the action of air, the extract is bitter, and that, when sweet, the medical efficacy of the remedy is impaired. It may be given in doses of half a drachm, or more, four or six times a day, dissolved in some aromatic water—a form preferable to that of pill. It may safely be prescribed as an alterative in cutaneous affections, and in those derangements of general health which are accompanied by obscure hepatic symptoms, and in which the usual treatment is ineffectual. Taraxacum is thought well of by several foreign writers of eminence, and is by them generally recommended in the form of liquid extract, or, as it is sometimes termed, *Mellago Taraxaci*; the expressed juice of the fresh root is also used in the dose of two fluid ounces every morning, with an equal quantity of milk.

According to John, the juice of taraxacum contains bitter extractive, caoutchouc, traces of resin, sugar, gum, a free acid, and sulphate, muriate, and phosphate of potassa and lime. For the following particulars respecting this root and its extract, I am indebted to Mr. Squire:—

"Fresh taraxacum root, when crushed and submitted to pressure, varies exceedingly, even in the same week, in the produce of extract, without any material difference being discernible in the root itself; and the average results of each month, taken separately, show a marked difference in the strength of the juice at different seasons. In the winter months, when it should be dug up for medicinal use, the fresh root loses, on drying, 75 per cent. of water. This root, washed, crushed, and pressed, will yield half its weight of dark juice, which coagulates, and becomes of a fawn colour. It yields, on evaporation, 25 per cent. of extract; but, if the expressed roots be further digested, more extract is obtained.

At different seasons of the year, one pound of extract is afforded by the following proportions of the expressed juice, namely:

January and February, 4 to 5 lbs. of juice = 1 lb. of extract.

March, 6 to 7 lbs. of juice = 1 lb. of extract.

April, May, 8 to 9 lbs. of juice = 1 lb. of extract, and during these months the juice is so aqueous, that it does not coadjulate spontaneously, as it does during the preceding months.

June, July, August, 6 to 7 lbs. of juice = 1 lb. of extract, and now it again coadjulates; the old roots are spongy, and the new ones very slender.

In September and October, 4 to 5 lbs. of juice = 1 lb. of extract.

In November and December, 4 lbs. of juice = 1 lb. of extract.

During November and December the root is in the most vigorous condition, and most abundant in those ingredients upon which its medicinal powers depend. Frost has a singular effect upon the growing roots, causing the bitterness to decrease, and sweetness to take its place; it is also observable that, on the disappearance of the frost, the bitter returns in a stronger degree, and the sweetness disappears.

The *dark* extract of the shops owes its sweetness to a curious change in the juice during evaporation; and if this process be much protracted, acetic acid is formed, which imparts to the extract a sensible acidity. When carefully prepared, extract of taraxacum is of a brown color, has a sensibly bitter taste, and a peculiar aroma, but it is not so sweet.

From the chemical examination which Mr. Squire has made of the expressed juice of the root of taraxacum, it appears to contain gum, albumen and gluten, an odorous principle, extractive, and a peculiar crystallizable bitter principle soluble in alcohol and water.—*Ib. from Brande's Dic. Mat. Med*, 1839.

---

*Hospitals in Holland.*—There are three hospitals in the Hague, one of which is civil, and the other two are military. The civil hospital is situated on the Zuid-wall, or south boulevard, in the neighbourhood of a flat and marshy country, resembling the fens of Lincolnshire. It is a neat brick building, capable of containing about two hundred patients; at present there is scarcely a fourth of that number. A stranger cannot obtain admittance without the permission of the medical man who constantly resides at the hospital, besides which a small donation is expected by the porter from each visitor. There are separate wards, (all small and extremely clean,) for medical, surgical, and obstetrical cases. The Jews occupy distinct wards, with attendants belonging to their own persuasion, in conformity with certain religious principles. There is a small and neat bath-room in the establishment. One case was shown to me in the surgical wards wherein there had been considerable inflammation of both corneæ, and had received the usual antiphlogistic treatment, with a seton in the arm. The method of coercion, adopted in a case of traumatic delirium, was by means of leathern straps, instead of a strait waistcoat. The principal strap was placed round the patient, close under the axillæ, and fastened from behind to the head of the bed. If the patient became very violent, the arms were confined at the wrist, close to the chest, by smaller additional straps attached to the larger strap, which encircled the body. In the medical wards three cases were pointed out to me—one of continued fever, which I was informed had been treated merely by lavements, and was doing well; the others were dropsy and cancer of the womb.

The obstetrical wards are divided into two departments; one smaller room for the actual delivery of the patients; the other for their reception after parturition. No contagious diseases are admitted in the hospital; there is, however, a separate building in progress, placed in the garden, for the reception of scarlet-fever, small-pox, measles, &c., which will contain four wards, two being intended for Jews. The garden is ornamented with an avenue of trees and flowers. The names of the physician and surgeon are Dr. Van de Watering and M. Wachter.

*Military Hospitals.*—There are two hospitals for soldiers at the Hague, the larger of which is termed the Military Hospital; the other, "Willem's Hospital," or hospital of the Princess of Orange.

*The Military Hospital.*—A stranger on applying to the porter of the military hospital for admission, is referred to the apothecary, who resides in the house adjoining, where he will readily obtain admittance, and a conductor to show the interior. The building is situated in the Burg-wall, and contains at present about one hundred and fifty patients; but when the new part is completed it will accommodate a great many more. There are separate wards for medical and surgical cases; and of the latter, the syphilitic and ophthalmic patients have wards exclusively to themselves. Ophthalmia is very prevalent. I was informed that there were two surgeons attached to the hospital, a surgeon-major, and one of the second class, who attended twice daily.

*The Hospital of the Princess of Orange* is situated in the Hekkelaan. It is fronted by a high wall inclosing a neat garden, and contains scarcely any patients at present, although large enough to hold a hundred persons.

*Medical Remuneration.*—The usual fee for the visit of a physician is a guilder or florin, which equals one shilling and eight-pence in English money. Several of the poorer tradespeople at the Hague subscribe a very small sum annually, to remunerate a particular medical man, and each family receives his attendance during the continuance of this subscription.

*License to practise.*—After a physician has received his diploma from one of the universities in Holland, if he wishes to practise, the diploma must be examined and signed by a person appointed for that purpose over each state and district in the country.—*Ib.*

---

*The Dublin Hospital Reports.*—A new volume of this important work is announced as being in course of preparation. On the high and justly earned character of the Dublin Hospital Reports, it is unnecessary for us to make any observation: for many years previous to the publication of the Dublin Medical Journal, this work and the Transactions of the Association of the College of Physicians were the only medical works emanating from Dublin in which reports and essays on surgical and medical subjects could be published.

*Dublin Journal.*

